# “We Need to Translate Research Into Meaningful HTLV Health Policies and Programs”: Webinar HTLV World Day 2021

**DOI:** 10.3389/fpubh.2022.883080

**Published:** 2022-05-10

**Authors:** Carolina Rosadas, Tatiane Assone, Leandro Sereno, Angelica Espinosa Miranda, Rubén Mayorga-Sagastume, Marcelo A. Freitas, Graham P. Taylor, Ricardo Ishak

**Affiliations:** ^1^Section of Virology, Department of Infectious Disease, Imperial College London, London, United Kingdom; ^2^HTLV Channel, Brazilia, Brazil; ^3^Faculty of Medicine, São Paulo University (USP), São Paulo, Brazil; ^4^Pan American Health Organization/World Health Organization, Washington, DC, United States; ^5^Departamento de Condições Crônicas e Infecções Sexualmente Transmissíveis, Secretaria de Vigilância em Saúde, Ministério da Saúde, Brasília, Brazil; ^6^Departamento de Medicina Social, Universidade Federal do Espírito Santo, Vitoria, Brazil; ^7^National Centre for Human Retrovirology, St Mary's Hospital, London, United Kingdom; ^8^Laboratório de Virologia, Universidade Federal do Pará, Pará, Brazil

**Keywords:** HTLV, prevention, elimination, health policies, epidemiology, disease, sexually transmitted infection, vertical transmission

## Introduction

Human T-cell lymphotropic virus type 1 (HTLV-1) is a neglected retrovirus that causes severe diseases worldwide. It is a blood borne virus that can also be transmitted by unprotected sex and from mother-to-child, predominantly *via* breastfeeding. In 2019 the International Retrovirology Association (IRVA) established November 10th as HTLV World Day with the aim to increase awareness about this infection. In 2020, HTLV Channel was founded with the same primary objective. HTLV Channel is an interactive platform that uses social media to disseminate research advances and basic knowledge about HTLV. In addition, this platform aims to support patients and to foster research collaboration, mainly in Latin America. In the year of its foundation, HTLV Channel celebrated the World HTLV Day with a webinar co-organized with the Brazilian Ministry of Health. In 2021, the Pan American Health Organization/World Health Organization (PAHO/WHO) joined forces with HTLV Channel to produce the HTLV World Day 2021 Webinar. Four decades after the discovery of HTLV (1, 2) the implementation of policies to prevent its transmission and for patient care remain limited (3). The Webinar entitled: “World HTLV Day 2021: International health policy forum for the elimination of HTLV—Advancing HTLV health policies around the world” aimed to discuss public policies toward HTLV-1/2, to identify barriers and opportunities that can impact the development of public health responses to this virus. This manuscript aims to reflect on some important points that were discussed during the meeting (available at https://youtu.be/pbpruvW5JA0 and https://youtu.be/g6DUXoqIH9A).

## The Impact of HTLV-1 Beyond HAM and ATL

“*We should be ashamed because we are still with these diseases. We are supporting health inequities. So, we should do something as a world community”* Prof. Patricia Garcia.

Although the amount of research compared with other diseases is small, most HTLV clinical research has been on HTLV-1-associated myelopathy (HAM) and adult T cell leukemia lymphoma (ATL), despite which patient care has improved little and both diseases still have poor outcome. The causative link between HTLV-1 and a multitude of inflammatory conditions, e.g., infective dermatitis, uveitis, pulmonary disease, and its impact on co-infections is starting to be better recognized and this was clearly presented at the Webinar by Dr. Fabiola Martin and Prof. Eduardo Gotuzzo. Indeed, the association of HTLV-1 with a 57% increase in the adjusted mortality rate revealed in a recent meta-analysis (4) significantly alters the previous widespread perception that this virus only impacts health of a very small number of those infected. In addition to these under recognized health impacts, the webinar discussed the little measured socioeconomic impact of HTLV-1 infection and the role of this virus in supporting inequities.

HTLV-1 infects mainly those with low-income, limited formal education, females, and vulnerable groups. HTLV-1-associated diseases and the stigma that is commonly associated with this infection impact patients' work capacity, reducing their income and accentuating health inequities (5, 6). Family aggregation aggravates this scenario (7). In fact, during the Webinar, patients' representatives clearly exemplified this difficult situation with their own experiences. Adjeane Oliveira, from Brazil, reported her early retirement due to HAM while Ema Moyano from Argentina, addressed family aggregation and the loss of family members due to HTLV-1-associated diseases (8). The negative impact of HTLV-1 is not restricted to patients' health, the loss of their loved ones or their income. The stigma of having an incurable sexually transmitted infection (STI) (9), sometimes accentuated by the disability caused by HAM, frequently results in what patients called “social death”. This is a difficult reality that needs to be considered when planning policies to HTLV-1/2 and when performing cost-effectiveness evaluations.

## There is a Need to Increase HTLV Testing Worldwide

“*If we do not look, we will not find it”* Dr. Noreen Jack.

HTLV-1/2 infection is considered a silent infection. Most patients are still considered asymptomatic, despite the clear impact of HTLV-1 on quality of life, mental health, and socio-economic status of those infected individuals who have not developed HAM or ATL. These two well characterized diseases that are associated with HTLV-1, HAM and ATLL, have a long incubation time, and their onset is usually late in adulthood. Therefore, there is a need for active surveillance to identify those infected. Until widespread screening is available targeted testing should be offered and include sexual partners and family members of PLHTLV, and those at higher risk of acquiring STIs and blood borne viral infection, such as patients with STIs, those that take Pre-Exposure Prophylaxis (PrEP) to prevent HIV infection, sex workers, and people who inject recreational drugs.

Most countries screen blood donors for HTLV-1/2 infection. In the Americas, the uptake of this policy is high. In 2016 and 2017, 90% of blood units collected in Latin America and the Caribbean were screened for HTLV-1/2 (10). However, in most cases, no confirmatory test is performed, and counseling is scant or absent. This represents missed opportunities to prevent new infections and to provide adequate care. In addition, data from blood banks are rarely used to provide epidemiological information for policymakers, despite the lack of current HTLV prevalence data for many countries in the region. In the USA, data from blood donors informs about HTLV-1/2 surveillance in the country. Reporting and analyzing data from blood banks would be a good starting point, acknowledging that it may be an under estimation of the real burden of HTLV-1/2 infection. Disease Registers for HTLV, such as the one established in the UK in 2004 are also useful to provide guidance for policymakers but have not been widely implemented.

The development of a low-cost, rapid, point of care test for HTLV-1/2 screening would facilitate test uptake worldwide and should be a research priority. Local and international agencies should establish guidelines for the diagnosis of HTLV infection, including the need of confirmatory testing.

## HTLV Awareness is Essential and Catalytical for the Advance of Health Policies

“*The lack of knowledge about HTLV is the main complicating factor”* Ms Adjeane Oliveira.

The lack of awareness about HTLV-1/2, including among health care workers, was mentioned by patients' representatives and experts as a major barrier to the advance of policies to control this infection. The lack of awareness prevents adequate care, contributes to increase stigma, and impairs the inclusion of HTLV-1/2 in the international and local health agenda. In addition, it is detrimental to research and contributes to the limited interest of pharmaceutical companies into the development of new therapeutic and preventive interventions and the scarcity of research funding in general.

The global community should take advantage of online platforms and social media to disseminate information about HTLV-1/2. In fact, the Webinar itself is a good example of how these tools may be useful to push for action. In Brazil, HTLV was included in an online course about STI for healthcare workers and a clinical guideline about HTLV was published. Similar guidance is under development in the UK and expected to be published during 2022. The HTLV Community should take this opportunity of renewed interest in HTLV-1/2 being shown by international agencies, such as the PAHO/WHO, to strengthen the discussion about this virus. In this regard, a strong collaboration between researchers, patients' representatives, and policy makers, as reported in Brazil and UK, is key to the implementation of effective measures to prevent and control HTLV-1.

It was interesting to observe the dichotomy when comparing the view of Australia's healthcare professionals and that of the aboriginal community from Central Australia, who suffer with a high prevalence of HTLV-1 infection, affecting almost half of the adult population (11). According to healthcare workers‘ perspective, it is not important to increase awareness about HTLV-1/2 among aboriginals in Central Australia and this information could be harmful for them. Those professionals assumed that aboriginal people would not understand about HTLV-1/2 and this was not a priority for them. On the contrary, people from these communities reinforced the importance to talk about HTLV-1/2 and how education is important to empower this population that is most affected by this virus and to prevent new infections (12). Although the HTLV situation in the Aboriginal communities of Central Australia represents a very particular scenario, this dichotomy is a barrier that is shared by many other settings. In fact, a similar argument was used by the antenatal screening committee from the UK when they decided not to implement HTLV-1/2 antenatal screening in the country. One of the arguments was that testing for HTLV-1/2 would be harmful for pregnant women as it could be stressful for them to be tested for an incurable infection. However, women‘s perspective was not assessed, and their views were not taken into account when deciding about this important policy. This highlights the importance of engaging with patient's representatives when designing policies toward HTLV-1/2 and the urgent need for research in this area, both of those who are already affected by HTLV-1/2 infection and those who would be screened. HTLV-1/2 antenatal screening was unanimously considered a major priority by all patients' representative and specialists participating in the Webinar.

## Integration of HTLV Into Existing Programs

“*We certainly can do better to reduce mortality and morbidity”* Prof. Peter Figueroa.

An opportunity to introduce policies for HTLV-1/2 at relatively low cost is to take advantage of programs that are already implemented. Most countries have local programs related to maternal health, HIV and STIs, neglected diseases, etc. PAHO/WHO recently launched a platform for the elimination of a number of infectious diseases (13). HTLV-1/2 could be integrated into these programs and use their established network, working capacity, knowledge, and experience. In Brazil, the inclusion of HTLV-1/2 into the department of chronic diseases and sexually transmitted infections was beneficial to the advances that were recently observed. In fact, most of the successful outcomes in that country resulted from the addition of HTLV-1/2 in the list of STIs. Patients' care can also be integrated into the response to sexually transmitted infections and sexual health more broadly. In the UK the National Center for Human Retrovirology (NCHR), is part of the Department of Sexual Health and HIV, at St Mary‘s Hospital and has been a model of patient‘s care.

It is important for policies to be tailored to the local setting. In this regard, particularities of indigenous communities from Brazil and Australia were shared during the Webinar. As stated above, the medical leadership‘s assumptions have been a major barrier to the implementation of policies for the aboriginal community in Australia. In Brazil, cultural, linguistic, and geographic barriers were identified as difficulties to implement policies targeting indigenous people in that country. In Brazil, the collaboration between HTLV-1/2 specialist researchers and the Special Secretary of Indigenous Health (SESAI) to increase awareness about HTLV-1/2 and the production of informative factsheets about HTLV-1/2 in different indigenous languages are examples of good practices that can be achieved at relatively low cost.

## Prevention of Mother to Child Transmission is a Major Priority

“*We need now to institute simple programs, starting with antenatal testing and mother-to-child transmission prevention” Prof. Peter Figueroa*.

There is a common belief that as there is no cure for HTLV infection, nothing should be done. However, the absence of a curative treatment for HTLV-1/2 infection reinforces the importance of implementing policies to prevent new infections. Several effective measures to prevent new infections are known. The effectiveness of avoidance or shortening the duration of breastfeeding to reduce HTLV-1 transmission is well recognized. The implementation of antenatal screening was unanimously identified as a priority. Indeed mother-to-child transmission disproportionally contributes to HTLV-1 associated diseases (14).

The implementation of HTLV-1/2 antenatal screening integrated with other maternal and child health programs is an opportunity to identify those infected, to prevent new transmissions, to obtain epidemiological data and to verify the success of the policy. For this to succeed there is a need to increase awareness about HTLV among healthcare professionals, so they can provide proper counseling for seropositive women.

## The Time to Act is Now

“*Why is this virus still around and without effective surveillance, control or cure?”* Dr. Fabiola Martin.

Professor Peter Figueroa's statement, reflected in the title of this manuscript “We need to translate research into meaningful health policies and programs to HTLV” summarizes the main message of the webinar. There is still a long way to go before we can start planning HTLV-1/2 elimination, but we must start paving the way now. Many different actors are involved: local governments, HTLV experts, patients' representatives and international agencies and need to start immediately to act together toward this goal. Paraphrasing a Chinese proverb: “The best time to act was decades ago. The second-best time is now.”

## Conclusion

**Figure 1:**
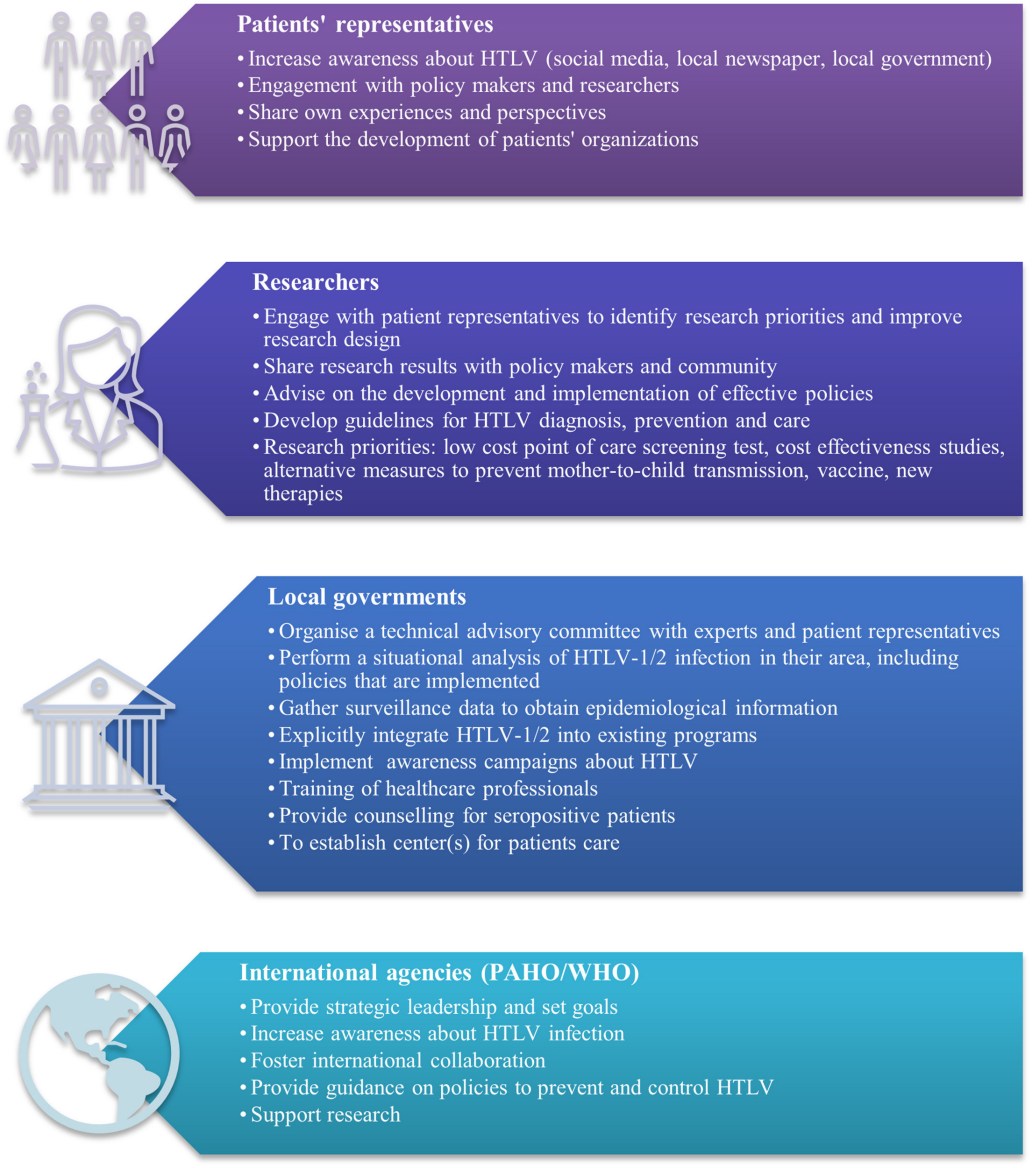
Short-term goals for an effective public health response to HTLV-1/2.

The short-term goals, for each of the actors, drawn from this interactive webinar are presented in the Figure 1. We hope that PAHO/WHO renewed interesting in HTLV-1 and that the HTLV World Day Webinar 2021 will be catalytic for an effective global response to HTLV-1/2.

## Author Contributions

CR, TA, LS, and RI conceptualized the manuscript. CR drafted the initial manuscript. CR, TA, LS, AM, RM-S, MAF, GPT, and RI wrote and critically revised the final manuscript. All authors approved the final version of the manuscript.

## Funding

RI is a Research Grantee (#312979/2018-5) of the Conselho Nacional de Desenvolvimento Cientifico e Tecnologico, CNPq. GPT is supported by NIHR Imperial College Trust. TA is supported by a scholarship from HCFMUSP with funds donated by NUBANK under the #HCCOMVIDA scheme.

## Conflict of Interest

CR and TA are co-founders of HTLV Channel, a non-profit self-funded platform by HTLV Channel. The remaining authors declare that the research was conducted in the absence of any commercial or financial relationships that could be construed as a potential conflict of interest.

## Publisher's Note

All claims expressed in this article are solely those of the authors and do not necessarily represent those of their affiliated organizations, or those of the publisher, the editors and the reviewers. Any product that may be evaluated in this article, or claim that may be made by its manufacturer, is not guaranteed or endorsed by the publisher.
